# A Clinical Treatise on the Diseases of the Nervous System

**Published:** 1879-08

**Authors:** 


A Clinical Treatise on the Diseases of the Nervous System. By M. Rosenthal, Prof. of Diseases of the Nervous System at Vienna, with a Preface by Prof. Charcot. Translated from the author's Revised and Enlarged Edition by L. Pretzel, M.D., Physician to the Class for Nervous Diseases, Bellevue Hospital, out door Department, etc. Wm. Wood & Co., Publishers, New York. 1879.

This is another issue of the valuable series of "Wood's Library of Standard Authors," which will add no little to the encyclopedic value of the volumes issued before. Prof. Charcot asserts in the preface to this work that the author has avoided the modern wide-spread penchant of dealing almost exclusively in physiological theorizing in treatises on these subjects, and has confined himself to the clinical and pathological details of nervous diseases. This is a very important innovation, and renders the work of much more practical value than it otherwise might be.

In the introductory part is given the general characteristics of cerebral affections, or a curtailed summary of the symptomatology of cerebral diseases. The subjects are divided into three classes, in which the brain, medulla oblongata and spinal column receive each their due consideration. The subdivisions consist in descriptions of 1. Diseases of the cranial meninges; 2. Of the cranial parenchyma; 3. Of the medulla oblongata; 4. Of the spinal meninges; 5. Of the parenchyma of the cord.

The affections under these various heads are treated of from a clinical standpoint, which renders the work very desirable to those making the nervous system their study.

